# Sex Differences in Age-Associated Rate of Decline in Grip Strength When Engaging in Vigorous Physical Activity

**DOI:** 10.3390/ijerph191711009

**Published:** 2022-09-02

**Authors:** Marianne Huebner, Frank Lawrence, Lara Lusa

**Affiliations:** 1 Department of Statistics and Probability, Michigan State University, East Lansing, MI 48824, USA; 2 Center for Statistical Training and Consulting, Michigan State University, East Lansing, MI 48824, USA; 3 Department of Mathematics, Natural Sciences and Technology, University of Primorska, 6000 Koper, Slovenia; 4 Institute for Biostatistics and Medical Informatics, Faculty of Medicine, University of Ljubljana, 1000 Ljubljana, Slovenia

**Keywords:** sport, exercise, aging, accelerated decline, body mass, menopause

## Abstract

Handgrip strength (GS) is used as an indicator of overall muscle strength and health outcomes for aging adults. GS has also been evaluated as a potential link with sport performances. We quantified the age-associated decline in grip strength for males and females engaged in weekly vigorous physical activity, differentiated by body mass, and investigated whether there was an acceleration of decline at any age. The Survey of Health, Ageing and Retirement in Europe is a multinational complex panel data survey with a target population of individuals aged 50 years or older. Data from 48,070 individuals from 20 European countries, collected from 2004 to 2015, were used in multivariable regression models to study the association of age and body weight with grip strength for individuals engaged in vigorous physical activity at least once a week. The annual rate of change in GS differed for males and females; it was constant from ages 50 to 55 years and then accelerated for females, possibly due to the menopausal transition. In contrast, the decline in GS accelerates with each year of increase in age for males. Higher body mass was associated with an increase in GS, but the increase was less pronounced for older males. The increase in GS diminished with a body mass above the median even with engagement in weekly vigorous physical activities. GS reference values for individuals engaged in vigorous physical activity add to existing reference values for general populations.

## 1. Introduction

Muscle strength is an indicator of health and fitness and declines with increasing age [1,2]. Grip strength (GS), in particular, has been used as a surrogate of overall muscle strength as it correlates with strength not only in the upper extremities but also with other muscle groups [3,4,5]. Declines in GS have been associated in many studies with functional disabilities, morbidity, or mortality, and GS has been deemed an important biomarker [3,6]. Decreasing physical activity appears to be a key factor involved in producing low muscle strength [7]. Previous studies reported associations of GS with aerobic capacity [8], health and fitness scores [9], and physical activity levels [10,11]. GS has been evaluated as a potential link with sport performances in several disciplines such as racket sports or ball sports [12]. While this has been studied in younger populations (under 20 or under 30 years of age), the decline in GS in older athletes has not been considered. GS starts to decline in the third decade of life [13,14,15] and the decline may accelerate with increasing age [16].

Lifestyle choices such as the regular practice of physical activity can improve GS [10,17]. Guidelines by the World Health Organization recommend 75–150 min per week of vigorous physical activity for adults or 150–300 min of moderate-intensity physical activity to decrease the risk of cardiovascular disease and enhance functional capacity [18].

Higher body mass is associated with better performance in strength sports and many models exist to normalize athletes’ performances [19,20]. Such normalization has limitations since the impact of body mass on performance may change with age. This has been examined in Olympic-style weightlifting [21] but very little is known about body mass and age interactions on other sport performances or on grip strength measures.

It has been shown that females have lower GS than males at all ages [13,14]. It is unclear whether sex-specific factors influence not only the strength but also the rate of performance decline. Menopause may be one such factor since physical performance begins to decline midlife in women at a faster rate in GS [22], weightlifting [23], or track and field disciplines that require explosive power [24]. The age-associated decline in powerlifting is less steep compared to Olympic-style weightlifting [25]. It is unclear whether there are sex differences in the rate of decline in strength (such as GS) that has been seen in explosive strength sports (such as Olympic-style weightlifting) and whether the age-associated decline in strength is constant or accelerates at older ages. The SHARE data provide a unique opportunity to examine the potential non-linearity association in more detail. While this is a population cohort, individuals engaged in vigorous physical activity could serve as a comparison group to athletes.

The objectives of our cross-sectional study were to quantify (i) the age-associated rate of decline in grip strength in adults 50 years and older engaged in vigorous physical activities at least once a week, (ii) sex differences in the rate of decline, (iii) age-related changes in the association of body mass with grip strength, and (iv) examining regional differences was a secondary aim as this has been observed in prior studies [13]. This was achieved by analyzing data from a large European survey and physical measurements in individuals ages 50 to 80 years from 20 countries. We hypothesized that there are age differences in the rate of decline in GS for women possibly due to hormonal changes during the transition into menopause. Furthermore, we hypothesized that body mass has diminishing returns for an increase in GS in this population engaged in weekly vigorous physical activities and that the increase in GS corresponding to higher body mass may be less steep at older ages.

## 2. Materials and Methods

### 2.1. Study Population

**Figure A1:**
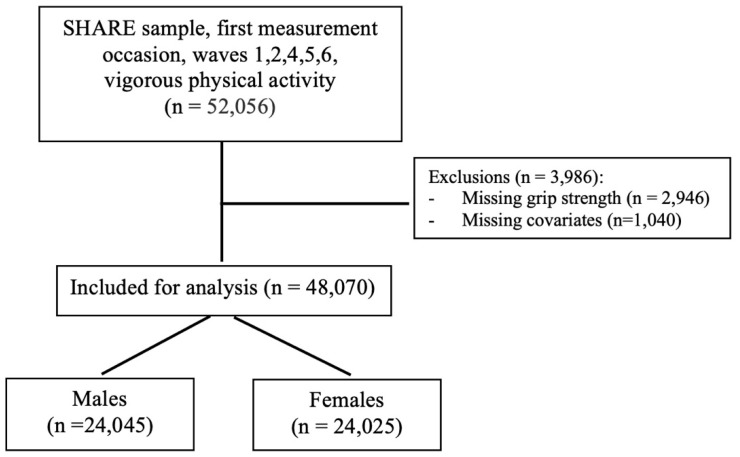
Study flow diagram.

The Survey of Health, Ageing and Retirement in Europe (SHARE) is a multinational complex panel data survey [26]. The target population includes all individuals who are at least 50 years old, speak the official language of the country, do not live abroad or in an institution such as a prison. From 2004 to 2015 (waves 1 through 7 without SHARELIFE) interviews were conducted in 20 European countries (http://www.share-project.org, Release 7.01, accessed on 15 July 2021). Countries that contributed data were Austria, Belgium, France, Germany, Ireland, Luxembourg, The Netherlands, and Switzerland (Western region), Italy, Greece, Spain, and Portugal (Southern), Denmark and Sweden (Northern), Croatia, Czech Republic, Estonia, Hungary, Poland, and Slovenia (Eastern) [14]. Inclusion criteria were ages 50 to 80 and engagement in vigorous physical activity at least once a week. The dataset included 48,070 individuals with valid GS measures at their baseline interview after data cleaning was performed [27] (Figure A1). Data cleaning involved tasks such as checking plausibility limits and consistency of values. We provided reproducible R code to efficiently work with SHARE data [28].

### 2.2. Measures

GS was measured using a handheld dynamometer (Smedley, S Dynamometer, TTM, Tokyo, 100 kg) with the elbow at a 90° angle. Two values were recorded for each hand alternating between left and right hand. Valid measurements were defined as the values of two measurements in one hand that differed by less than 20 kg [13]. The maximum GS was determined from these repetitions. Participants self-reported vigorous physical activity (in response to the question “How often do you engage in vigorous physical activity, such as sports, heavy housework, or a job that involves physical labour?”) with response options: (i) more than once a week, (ii) once a week, (iii) up to three times a month and (iv) hardly ever or never. Self-reported height, body mass, education level per International Standard Classification of Education (ISCED), and current smoking were also considered. These are factors that could potentially confound the association of age with GS identified in previous studies [13].

### 2.3. Statistical Analysis

Separate models were constructed for males and females engaged in vigorous physical activity at least once a week. A multivariable regression model was used to estimate the association of age, body mass, and height with GS. The models were adjusted for current smoking status (yes or no), education level (high (ISCED 5 or 6) vs. medium or low), and regions (Northern, Southern, Eastern, Western). Data cleaning and mitigating missing data in covariates was previously described [28]. A complete case analysis with a total of 48,070 individuals (24,025 females and 24,045 males) was performed, since missingness in any variables was limited to 6.6% for females and 5.6% for males. Multivariable regression models with linear, quadratic, and cubic powers were compared for age, body weight, and height, and likelihood ratio tests were used to select cubic power as the appropriate choice for age and body weight. Quadratic or cubic power for body weight is often used in performance normalization models in strength sports [19]. Some variables were rescaled to stabilize the estimation of model parameters [29]. Age was centered at 50 years and rescaled by 10. The centering was performed at age 50 because that was the age of the youngest participant. Centering at that age ensures that the estimated intercept is within the range of the data. It is accomplished by taking each participant’s age and subtracting 50. The centered age is divided by 10, thus a unit change in the model estimated is interpreted as a change of 10 years. Body mass and height were centered at the median at age 50, separately for females and males. Predicted values of GS were calculated from the regression models for non-smoking individuals of median height at age 50 (176 cm and 164 cm for males and females, respectively). The rate of change is the first derivative of these predicted curves. Bootstrap resampling was used to estimate confidence intervals for the maximum of the rate of change in GS with age. A random sample of 24,000 subjects was drawn at each iteration, the regression model was refitted to obtain a new estimate and annual differences were calculated. The process was repeated 500 times for males and females, and confidence intervals were obtained for identifying the age of smallest decline in GS. All analyses were conducted with the statistical software R (version 4.0.3) [30]. *P*-values less than 0.05 were considered statistically significant.

## 3. Results

A total of 48,070 individuals were included in the analysis with 50% females and overall median age 60 (range 50 to 80). The characteristics are presented in Table 1.

**Figure 1:**
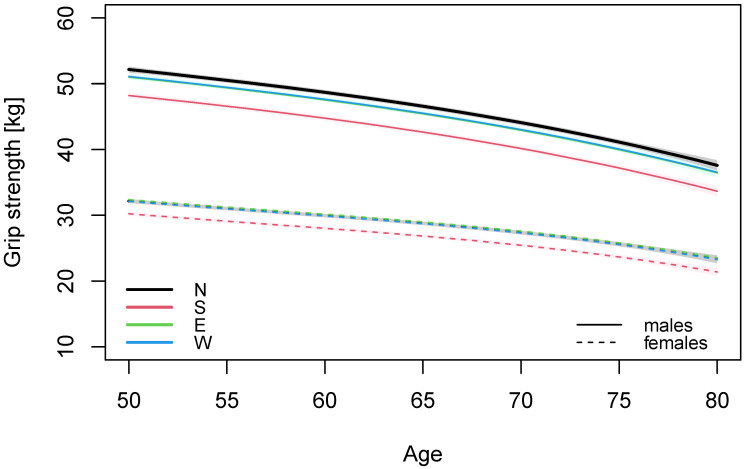
Predicted age-associated decline in grip strength for males and females with 95% confidence bands by region.

Grip strength was higher for males than for females (Figure 1). The average GS at age 50 years was 51.3 kg (95% CI: 51.0, 51.7) for males and 31.7 kg (95% CI: 31.5, 32.0) for females after adjusting for height, body mass, smoking, education level, and region (Table 2). Individuals from southern countries had lower GS than individuals from western countries (1.9 kg and 2.9 kg for females and males, respectively), but also were on average shorter in height. A 10 cm increase in height above the median improved GS by 2.3 and 2.0 kg for females and males, respectively.

Regional predicted GS values and 95% confidence intervals for individuals of median height and weight, non-smoking and with higher education level are shown in Table 3. Males living in Western European countries had higher GS than those from Southern European countries, at age 50 the estimated GS is 51.1 kg (95% CI: 50.7, 51.5) and 48.2 kg (95% CI: 47.8, 48.6), respectively (Table 3). The corresponding GS for females at age 50 were 32.1 kg (95% CI: 31.8, 32.4) for Western Europeans and 30.2 kg (95% CI: 29.9, 30.5) for Southern Europeans. Eastern and Western Europeans had similar GS, while Northern European males were stronger.

In our study, the loss in GS in Northern European countries corresponded to 3.5 kg and 2.3 kg from ages 50 to 60 for men and women, respectively, while from ages 70 to 80 the loss was 6.5 kg and 4.1 kg, respectively (Table 3).

**Figure 2:**
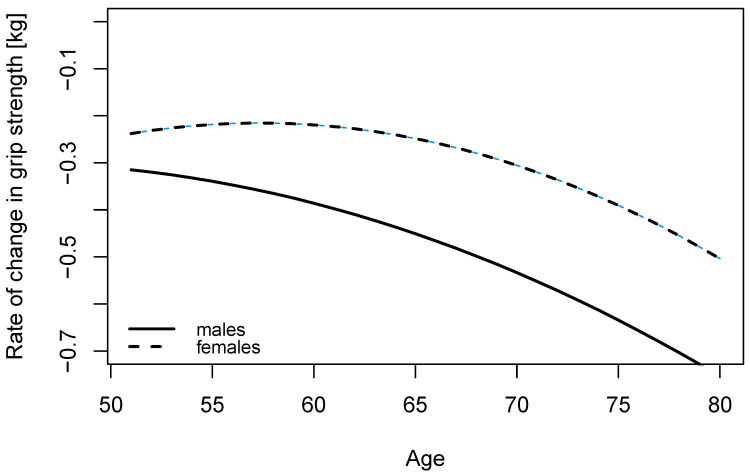
Rate of change in grip strength by age for males and females at median height and weight, engaged in vigorous physical activity at least once a week.

Compared to age 50, GS was 93% at age 60 and 84% at age 70 for males. The corresponding percentages for females were 93% and 85%. The curve for females was undulating and the rate of change in GS was the same until about age 60 and then accelerated (Figure 2). A bootstrap estimate for the median change point was 55 years (95% CI: 50, 60). The estimated change point for males was 50 years (95% bootstrap confidence interval 50, 56) indicating a greater decline with each year of age.

**Figure 3:**
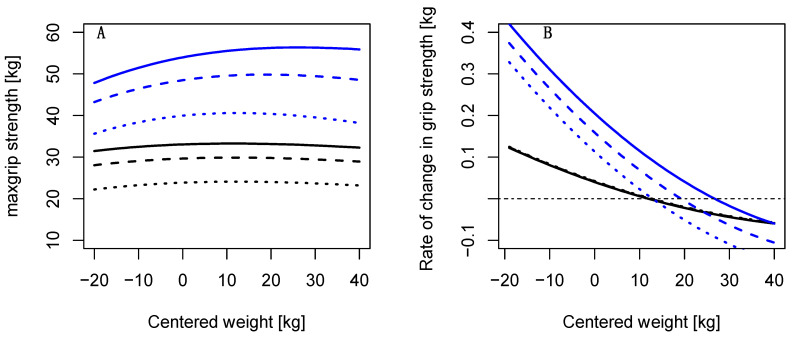
(**A**) Increase in grip strength by body mass (a centered weight of 0 refers to the mean body mass at age 50 (82 kg for males, 68 kg for females) shown at ages 50, 65, and 80. (**B**) Rate of change in grip strength by body mass (a centered weight of 0 refers to the mean body mass at age 50) shown at ages 50, 65, and 80. A caption on a single line should be centered.

A 1 kg increase in body mass from the median was associated with an increase of 0.06 kg and 0.18 kg and for females and males, respectively (Figure 3A). The increase was steeper for a body mass lower than the median, but decreased at higher body mass, approximately at 30 kg above the median body mass (Figure 3B). This was more pronounced for older males at ages 65 or 80 compared to males at age 50.

## 4. Discussion

The main objective of our cross-sectional study was to quantify the age-associated rate of decline in grip strength in adults 50 years and older engaged in vigorous physical activities at least once a week. We investigated sex differences in the rate of decline and age-related changes in association of body mass with grip strength. A total of 48,070 grip strength results from unique older individuals, ages 50 to 80 years, engaged in weekly vigorous physical activity, from 20 countries were analyzed. Females accounted for 50%. This is the first study that considered the annual rate of change in age-associated decline of GS for individuals engaged in at least weekly vigorous physical activities and the incremental difference for each additional kg in body mass. The main findings were as follows: (i) the rate of change differed for males and females; it was constant from ages 50 to 55 years and then accelerated for females, but declined faster for males; (ii) higher body mass was associated with an increase in GS, but the increase was less pronounced for older ages in males; and (iii) the increase in GS diminished with a body mass above the median even with engagement in weekly vigorous physical activity.

### 4.1. Sex Differences in the Age-Associated Decline in Grip Strength

GS declines at a faster rate at older ages, this was already considered by Samson et al. [16] who fitted linear regression models, separately for 74 women and 81 men, with a change point at age 55 to estimate the decline in GS before age 55 and a steeper decline after age 55. Our findings using polynomial regression models indicated that the decline in GS for women is at first constant and then accelerates at age 55 years, but the acceleration could begin anywhere between 50 and 60 (95% confidence interval: 50, 60). For men the decline was steeper with increasing age, accelerating with each year from age 50 years (95% confidence interval 50, 56), thus there may not be a change point. Our findings on the rate of change in GS with age is consistent with a study for performance decline in sports requiring explosive power such as Olympic-style weightlifting or throwing disciplines [23,24]. Compared to male weightlifters, females had a steeper performance decline than males starting in their 40s coinciding with a transition into menopause, and then the decline slowed. There is a substantial decrease in testosterone concentrations and estrogen during the first year of menopause. This is accompanied by accelerated decline in muscle mass and strength [22,31,32]. Post-menopausal women have lower GS compared to premenopausal women [33,34]. In female weightlifters, 74% reached natural menopause between 45 and 54 years, and 13% at 55 years or older [35]. Since the SHARE data consists of individuals ages 50 and an older when many women have already reached menopause, thus the change to accelerated decline could be explained by hormonal status. Due the scarcity of performance data of female weightlifters older than 60, it is not clear how the performance decline continues for older women weightlifters and thus our findings about GS decline could be helpful as an indicator. Anton et al. [25] applied regression models to pooled body mass data and concluded that female powerlifters, ages 35 and older, experienced a steeper decline than men. This differs from our finding that decline in GS in females is less steep than in males despite both performance measures being related to muscle strength. The differences seen in the powerlifting study are likely due to using American records and world records from 2002. Many more women have entered strength sports since then and performances have improved [36,37]. Strength sports such as powerlifting have a less steep performance decline than Olympic-style weightlifting that requires explosive strength [25]. Thus, it is expected that GS declines less steeply than this weightlifting. For example, the male Olympic-style weightlifting performances at age 60 are 83% of those at age 50 and reducing to 70% at age 70 [23]. In our study, male GS is 93% at age 60 of those at age 50, reducing to 84% at age 70, and 71% at age 80 years.

### 4.2. Nonlinearity in Decline of Grip Strength

Many studies dichotomized GS as an indicator for low GS to study associations with health outcomes [38,39]. However, the problems with dichotomization are well-known. It underestimates the extent of variation and increases the risk of false positives and conceals non-linearity [40]. Sociodemographic factors influence GS, and the decline is non-linear with age, thus cut points for low GS need to be adjusted for such populations. Our findings about the form of decline in GS differ from previous publications in several ways. Previous publications showed a linear decrease in GS [5,13,41]. Some studies pooled data in age groups and then applied linear models [14]. However, linear regression models with age as a covariate do not consider the rate of change of the decline and do not take hormonal life cycles of women into account. In a German study with 769 adult volunteers, Gunther et al. [15] considered polynomial functions of age and BMI in a group of ages 20 to 95. The final model included both sexes, cubic age, and BMI in the model. Regression models that include both sexes [15] allow for the estimation of sex difference in the same model but might overlook a different form of decline for males and females. In a Danish study of 8342 individuals, Frederickson et al. [5] estimated a GS with a linear regression model with age and height as independent variables. In the age span from 50 to 85, men and women had a loss in GS 5.9 kg and 3.1 kg per decade, respectively. In our study the loss in GS in Northern European countries (Denmark and Sweden) varied by decade of age corresponding to a decline of 3.5 kg and 2.3 kg from ages 50 to 60 for men and women, respectively, while from ages 70 to 80 the loss in grip strength was higher, 6.5 kg and 4.1 kg, respectively.

### 4.3. Body Mass and Grip Strength at Different Ages

The association of body mass and GS was discussed by Keevil et al. [42] who found that larger overall body mass, indicated by higher BMI, was associated with stronger GS but larger waist circumference was associated with lower GS. This corresponds to our findings that an increase in body mass is associated with an increase in GS. However, a body mass of more than 30 kg above the median weight was associated with a decrease in GS. Body mass has a smaller effect on GS at older ages. Such a differential impact of body mass was also seen in Olympic-style weightlifting where body mass had a smaller effect on performance at older ages than at younger ages [21,37].

### 4.4. Regional Differences in Grip Strength

Height was associated with GS. Male and female residents of Southern European countries (Greece, Italy, Portugal, and Spain) had a shorter stature and a lower GS compared to the same sex in other European regions. Male residents in Denmark and Sweden had a higher GS than males in Western or Eastern European regions. A North–South gradient was noted in other European studies [13,14,43]. However, the rate of decline did not differ between the regions.

### 4.5. Strengths and Limitations

A strength of this study is the participation of 20 countries and a large cross-sectional sample size sampled from community-dwelling populations engaged in vigorous physical activity. Although individuals engaged in regular vigorous physical activity is a broader group than athletes, our study addresses sociodemographic limitations of studies regarding older athletes who are primarily affluent and highly educated. This allows us to investigate the association of functional forms of age, height, and body mass with GS and contrast with findings from strength sports. There are several limitations. First, some SHARE survey information was self-reported, such as height and education level and this is a limitation. Second, while GS is widely used as a simple assessment of muscle strength, its value as a proxy for overall strength is limited due to only a moderate correlation with other strength measures such as knee extensor strength [44].

## 5. Conclusions

To conclude, we observed that the rate of decline in grip strength with age is nonlinear and accelerates at older ages. For females the rate of decline is constant after age 50 possibly due to reaching menopausal status before it accelerates at older ages. We provide reference values of grip strength for males and females engaged in vigorous physical activities in different regions. This may be helpful to health care providers and exercise physiologists as it adds information to existing reference values in general populations irrespective of physical activity. A clear understanding of the rate of decline in grip strength with increasing age and determinants for grip strength as a surrogate for muscle strength can provide insights into strength across the lifespan. A relevant implication is to encourage vigorous physical activities at all ages and possibly increasing the volume with increasing age [18], and to target weight management to slow the decline in grip strength and thus improve quality of life. Future research should address the influence of hormonal changes, such as menopause, on the decline in grip strength.

## Figures and Tables

**Table 1:** Baseline characteristics.

	Males *n* = 24,045	Females *n* = 24,025
Age [years]	60 (54, 67)	59 (54, 66)
Age group		
50–59	48% (11,539)	52% (12,433)
60–69	35% (8452)	33% (7991)
70–80	17% (4054)	15% (3601)
Body mass [kg]	82 (75, 90)	68 (60, 77)
Height [cm]	176 (171, 180)	164 (160, 168)
Education, high	25% (6100)	23% (5626)
Current smoker, yes	24% (5748)	19% (4450)
Region		
West	44% (10,554)	44% (10,504)
North	14% (3252)	13% (3109)
South	18% (4261)	18% (4240)
East	25% (5978)	26% (6172)

Continuous variables are summarized as median (first, third quartile) or percent (counts) as appropriate.

**Table 2:** Multivariable regression model results for males and females.

	Males			Females		
Variable	Estimate	95% CI	*p*-Value	Estimate	95% CI	*p*-Value
Intercept	51.315	(50.97, 51.66)	<0.001	31.717	(31.48, 31.96)	<0.001
Age [10 years]	−3.126	(−4.16, −2.09)	<0.001	−2.416	(−3.15, −1.68)	<0.001
Age ^2^	−0.214	(−1.13, 0.7)	0.646	0.383	(−0.28, 1.05)	0.258
Age ^3^	−0.120	(−0.34, 0.1)	0.288	−0.187	(−0.35, −0.02)	0.026
Weight [10 kg]	−0.187	(−0.35, −0.02)	<0.001	0.540	(0.43, 0.65)	<0.001
Weight ^2^	1.784	(1.62, 1.94)	<0.001	−0.132	(−0.17, −0.09)	<0.001
Weight ^3^	−0.303	(−0.36, −0.25)	<0.001	0.008	(0.002, 0.012)	0.002
Height [10 cm]	0.017	(0.01, 0.02)	0.002	2.290	(2.16, 2.42)	<0.001
Education, high	−0.221	(−0.46, 0.02)	0.066	0.393	(0.21, 0.57)	<0.001
Smoking, yes	−0.174	(−0.42, 0.07)	0.157	0.172	(−0.02, 0.36)	0.078
Region, South	−2.880	(−3.17, −2.59)	<0.001	−1.882	(−2.09, −1.67)	<0.001
Region, North	1.057	(0.75, 1.37)	<0.001	0.048	(−0.19, 0.28)	0.687
Region, East	−0.098	(−0.35, 0.16)	0.451	0.272	(0.09, 0.46)	0.004
Age × Weight	−0.029	(−0.04, −0.02)	<0.001	0.000	(−0.01, 0.01)	0.903

Age was centered at 50 years; weight and height were centered at the median value at age 50. Age ^2^, Age ^3^,Weight ^2^, Weight ^3^ refer to quadratic and cubic terms in the model, respectively.

**Table 3:** Grip strength reference values (mean, 95% confidence intervals) if engaged in weekly vigorous physical activity.

Age	Males				Females			
	North	South	East	West	North	South	East	West
50	52.2 (51.7, 52.6)	48.2 (47.8, 48.6)	51.0 (50.6, 51.4)	51.1 (50.7, 51.5)	32.2 (31.9, 32.5)	30.2 (29.9, 30.5)	32.4 (32.1, 32.7)	32.1 (31.8, 32.4)
55	50.5 (50.2, 50.9)	46.6 (46.2, 46.9)	49.4 (49, 49.7)	49.5 (49.2, 49.7)	31.0 (30.8, 31.3)	29.1 (28.8, 29.3)	31.2 (31, 31.5)	31.0 (30.8, 31.2)
60	48.7 (48.3, 49)	44.8 (44.4, 45.1)	47.5 (47.2, 47.8)	47.6 (47.4, 47.9)	29.9 (29.7, 30.2)	28.0 (27.8, 28.3)	30.2 (29.9, 30.4)	29.9 (29.7, 30.1)
65	46.6 (46.2, 46.9)	42.6 (42.3, 43)	45.4 (45.1, 45.7)	45.5 (45.3, 45.8)	28.8 (28.5, 29)	26.8 (26.6, 27.1)	29.0 (28.8, 29.2)	28.7 (28.5, 28.9)
70	44.1 (43.7, 44.4)	40.1 (39.8, 40.5)	42.9 (42.6, 43.3)	43 (42.7, 43.3)	27.4 (27.1, 27.6)	25.4 (25.2, 25.7)	27.6 (27.3, 27.8)	27.3 (27.1, 27.5)
75	41.1 (40.7, 41.5)	37.2 (36.8, 37.6)	40.0 (39.6, 40.3)	40.1 (39.7, 40.4)	25.6 (25.3, 25.9)	23.7 (23.3, 24)	25.8 (25.5, 26.1)	25.5 (25.3, 25.8)

Values are predicted from the regression models for non-smoking individuals of median height (176 cm and 164 cm for males and females, respectively) and median weight (82 kg and 68 kg for males and females, respectively) at age 50.

## Data Availability

This study used data from the Survey of Health, Ageing and Retirement in Europe, which is freely available to academic researchers. http://www.share-project.org, Release 7.0.1, accessed on 15 July 2021.
